# Size-controlled green synthesis of silver nanoparticles mediated by gum ghatti (*Anogeissus latifolia*) and its biological activity

**DOI:** 10.1186/2191-2858-2-17

**Published:** 2012-05-09

**Authors:** Aruna Jyothi Kora, Sashidhar Rao Beedu, Arunachalam Jayaraman

**Affiliations:** 1 National Centre for Compositional Characterisation of Materials (NCCCM), Bhabha Atomic Research Centre, ECIL PO, Hyderabad, 500 062, AP, India; 2 Department of Biochemistry, University College of Science, Osmania University, Hyderabad, 500 007,, AP, India

**Keywords:** Antibacterial, Autoclaving, Gum ghatti, Silver nanoparticles, Surface-Enhanced Raman Scattering (SERS)

## Abstract

**Background:**

Gum ghatti is a proteinaceous edible, exudate tree gum of India and is also used in traditional medicine. A facile and ecofriendly green method has been developed for the synthesis of silver nanoparticles from silver nitrate using gum ghatti (*Anogeissus latifolia*) as a reducing and stabilizing agent. The influence of concentration of gum and reaction time on the synthesis of nanoparticles was studied. UV–visible spectroscopy, transmission electron microscopy and X-ray diffraction analytical techniques were used to characterize the synthesized nanoparticles.

**Results:**

By optimizing the reaction conditions, we could achieve nearly monodispersed and size controlled spherical nanoparticles of around 5.7 ± 0.2 nm. A possible mechanism involved in the reduction and stabilization of nanoparticles has been investigated using Fourier transform infrared spectroscopy and Raman spectroscopy.

**Conclusions:**

The synthesized silver nanoparticles had significant antibacterial action on both the Gram classes of bacteria. As the silver nanoparticles are encapsulated with functional group rich gum, they can be easily integrated for various biological applications.

## Background

A survey of earlier literature suggests that various natural polymers such as starch [1], chitosan [2], and tannic acid [3] have been reported as reducing agents for the synthesis of silver and gold nanoparticles. It has been demonstrated that the plant-based exudate gums such as gum *Acacia*[4] and gum kondagogu [5] can be utilized as reducing and stabilizing agents for the silver nanoparticle biosynthesis. Gum gellan, a microbial heteropolysaccharide, was employed for similar purpose in the case of gold nanoparticles [6]. Gum ghatti is a naturally occurring water soluble, complex polysaccharide derived as an exudate from the bark of *Anogeissus latifolia* (Combretaceae family), a native tree of the Indian sub-continent. The name gum ghatti has originated from its transportation through mountain passes or ghats. This native Indian gum is collected from the forests by the tribals and marketed through government organizations such as Girijan Co-operative Corporation Ltd., Visakhapatnam, India. The world production of gum ghatti is about 1,000–1,500 MT/year [7,8]. This biopolymer is an arabinogalactan type of natural gum and its morphological, structural, physico-chemical, compositional, solution, thermal, rheological, and emulsifying properties have been well documented and studied [9-17]. This biopolymer is a high-arabinose, protein rich, acidic heteropolysaccharide, occurring in nature as mixed calcium, magnesium, potassium, and sodium salt [12-14,16]. The primary structure of this gum is composed of sugars such as, l-arabinose, d-galactose, d-mannose, d-xylose, and d-glucuronic acid in a molar ratio of 48:29:10:5:10 and *<* 1% of rhamnose, which is present as non-reducing end-groups. The gum contains alternating 4-*O-*substituted and 2-*O*-substituted *α*d-mannopyranose units and chains of 1 → 6 linked *β*d-galactopyranose units with side chains of l-arabinofuranose residues. Six percent of rhamnose in the polysaccharide is linked to the galactose backbone as *α*-Rhap-(1 → 4) β-galactopyranose side chain. It has a molecular weight of 8.94 × 10^7^ g/mol [12,13,15,16].

The gum ghatti with a CAS number 9000-28-6 is recognized as “generally recognized as safe” (GRAS) and approved as a food ingredient (Code 184.1333) by the Food and Drug Administration, USA, under the function of emulsifier and emulsifier salt. Its use in food is also approved in Japan, China, South Korea, Singapore, Russia, Australia, South Africa, Iran, Saudi Arabia, Latin America, and other countries. But, it is not approved as a food additive in European Union and not been accorded a European food safety E number. It is considered as a food grade additives of food by the Bureau of Indian Standards, India under Indian Standard IS 7239:1974 [13,15,16]. In India, the application of this hydrocolloid in traditional medicine and food preparations is well known for centuries. The gum is fed to the lactating mothers in the form of *laddu* to enhance the nutrients in milk as well as to prevent the post-delivery backache [18]. The gum *laddu* is also eaten as a heating agent during winter season [18,19]. The gum ghatti is comprised of around 80% soluble dietary fiber and acts a prebiotic by supplying the matrix required to sustain the bacterial flora of the human colon. This hydrocolloid is resistant to gastrointestinal enzymes and known to be degraded enzymatically only by the specific microflora of the colon such as *Bifidobacterium longum*, thereby aiding in bifidus fermentation [20-22]. This gum is also given for the treatment of diarrhea and diabetes [23]. Earlier studies on gum ghatti fed white leghorn cockerels and albino rats have established the hypolipidemic activity of gum ghatti [24,25]. Recent studies have established that gum ghatti has a potential application as a release modifier for controlled drug delivery [26]. Gum ghatti has long been used in non-food applications, such as, calico printing, explosives, varnishes, car polishes, ceramics, cosmetics; and in pharmaceutical, textile, paper, petroleum, and mining industries. Also, this biopolymer aids in various photoelectric determinations [7,8,13,16,23].

The attractive features of gum ghatti prompted us to use this biopolymer for the synthesis and stabilization of silver nanoparticles due to its (i) edible nature and GRAS [13]; (ii) natural availability and low cost [23]; (iii) intermediate viscosity between gum arabic and gum karaya [14,15]; (iv) greater stability to pH acidification, electrolyte addition, and high-pressure treatment [15,17]; (v) higher emulsification ability and superior emulsion storage stability at lower concentrations [15], and (vi) exceptional interfacial characteristics with faster kinetics [17]. The green synthesis of inherently safer silver nanoparticles depends on the adoption of the basic requirements of green chemistry; the solvent medium, the benign reducing agent, and the non-hazardous stabilizing agent [1,27]. In this context, we have explored and developed a facile and green synthetic route for the production of silver nanoparticles using a proteinaceous, edible, renewable natural plant polymer, gum ghatti as both the reducing and stabilizing agents. Being a natural polymer, gum ghatti is amenable for biodegradation. The synthesis was carried out in aqueous medium by autoclaving, without the addition of any external chemical reducing agent. In this study, autoclaving was adopted as a synthetic route to produce sterile silver nanoparticles that are completely free from bacteria, viruses, and spores, which would suit biological applications. The focus of this study was on (i) the synthesis, (ii) characterization, and (iii) capping and stabilization of silver nanoparticles. In addition, we have also demonstrated the antibacterial activity of the prepared nanoparticles on Gram-positive and Gram-negative bacteria for finding out the potential of the generated nanoparticles for various environmental and biomedical applications.

## Methods

### Characterization of synthesized silver nanoparticles

In order to study the formation of silver nanoparticles, the UV–Visible absorption spectra of the prepared colloidal solutions were recorded using an Elico SL 196 spectrophotometer (Hyderabad, India), from 250 to 800 nm, against autoclaved gum blank. The absorption spectra of gum before and after autoclaving were also recorded against ultra pure water blank. The size and shape of the nanoparticles were obtained with Hitachi H 7500 (Tokyo, Japan) and JEOL 3010 (Tokyo, Japan) transmission electron microscopes (TEM), operating at 80 and 200 kV, respectively. Samples were prepared by depositing a drop of colloidal solution on a carbon-coated copper grid and drying at room temperature. The X-ray diffraction (XRD) analysis was conducted with a Rigaku, Ultima IV diffractometer (Tokyo, Japan) using monochromatic Cu Kα radiation (λ = 1.5406 Å) running at 40 kV and 30 mA. The intensity data for the nanoparticle solution deposited on a glass slide were collected over a 2θ range of 35–85° with a scan rate of 1°/min. The nanoparticles were recovered from the synthesized solutions by centrifugation and made into powders using a FTS Systems, Dura-Dry^TM^ MP freeze dryer (New York, USA). The IR spectra of the lyophilized samples were recorded using a Bruker Optics, TENSOR 27 FT-IR spectrometer (Ettlingen, Germany); over a spectral range of 400–4000 cm^–1^. The Raman spectrum of the synthesized nanoparticles was recorded at room temperature using the 532-nm line from a SUWTECH, G-SLM diode laser (Shanghai, China). The scattered light was collected and detected using a CCD-based monochromator, covering a spectral range of 150–1700 cm^–1^. The sample solution was taken in a standard 1 cm × 1 cm cuvette and placed in the path of the laser beam.

## Results and discussion

### Synthesis of silver nanoparticles

The present experimental investigation reports the green synthesis of silver nanoparticles using gum ghatti by autoclaving. This method utilizes a proteinaceous, edible, renewable, and water soluble biopolymer; gum ghatti which functions as both reducing and stabilizing agents during synthesis. By virtue of being a natural polymer, this gum is also amenable for biodegradation. The process of autoclaving makes the silver nanoparticles intrinsically safe and sterile, in environmentally benign solvent water. Moreover, generation of gum–silver nanoparticles by autoclaving is a prerequisite for biological applications. Thus, the adopted method is meeting the requirements of green chemistry principles.

### Proposed mechanism of reduction

During autoclaving at 121°C under the influence of temperature and pressure (103 kPa), this biopolymer expands and becomes more accessible for the silver ions to interact with the available functional groups on the gum as observed earlier for starch [1]. The gum has been categorized under arabinogalactan due to the abundance of arabinose and galactose. This acidic heteropolysaccharide is known to be rich in uronic acid content and shows a pH of 4.5–5.5 [8,14-17]. The presence of hydroxyl and carboxylic groups on this biopolymer [28] facilitates the complexation of silver ions. Subsequently, these silver ions oxidize the hydroxyl groups to carbonyl groups, during which the silver ions are reduced to elemental silver. In addition to this inherent oxidation, the dissolved air may also causes oxidation of the existing hydroxyl groups to carbonyl groups such as aldehydes and carboxylates. In turn, these powerful reducing aldehyde groups along with the other existing carbonyl groups reduce more and more of silver ions to elemental silver. Further, these nanoparticles are probably capped and stabilized by the polysaccharides along with the proteins present in the gum. As these carbohydrate polymers are very complex, it is most likely that more than one mechanism is involved in the complexation and subsequent reduction of silver ions by gum ghatti during autoclaving. Silver ion complexation by hydroxyl groups and its subsequent reduction by aldehyde groups are reported for starch, in which silver nanoparticles were produced by autoclaving [1]. Silver nanoparticles produced using gum *Acacia*, carboxylate groups involving complexation of silver ions and its subsequent reduction by hydroxyl groups were reported [4].

The reduction of silver ions by this gum even at room temperature was observed. But, the formed nanoparticles were not stable and aggregated due to lack of stabilization of the synthesized nanoparticles. It was noticed that the autoclaving at 121°C and 103 kPa of pressure, increased the extent of synthesis and stabilization of the nanoparticles. It is known that elevated temperature and pressure accelerate the synthesis of nanoparticles [1]. Besides, this process complexly eliminates the microbial contamination possibly acquired during gum secretion, collection, handling, and transportation.

### Characterization of synthesized silver nanoparticles

#### UV–Visible spectroscopy

**Figure 1:**
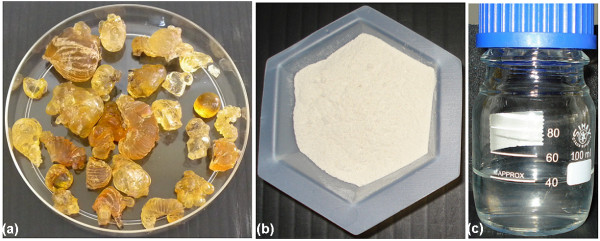
A digital photograph showing (a) Gum tears of grade 1 quality, (b) gum powder sieved to 38 μm particle size, and (c) centrifuged gum solution of 0.5% (w/v).

**Figure 2:**
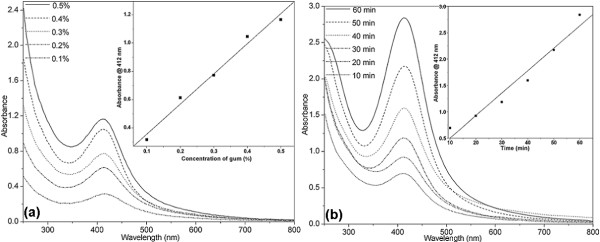
**The UV–Vis absorption spectra of silver nanoparticles synthesized: (a) by autoclaving different concentrations of gum ghatti solutions at 1 mM AgNO**_**3**_**concentration for 30 min; inset plot of A**_**max**_**versus gum concentration and (b) with 0.5% (w/v) gum ghatti solutions at 1 mM AgNO**_**3**_**concentration for different durations of autoclaving; inset plot of A**_**max**_**versus autoclaving time.**

The UV–Vis absorption spectroscopy is one of the most widely used simple and sensitive techniques for the observation of nanoparticle synthesis. In order to monitor the formation of silver nanoparticles, the absorption spectra of synthesized silver nanoparticles were recorded against respective autoclaved gum blanks. Figure 1 is indicating (a) gum tears of grade 1 quality, (b) gum powder sieved to 38 μm particle size, and (c) centrifuged gum solution of 0.5%. To optimize the nanoparticle synthesis, the influence of parameters such as concentration of gum and reaction time was studied. The role of gum concentration on the synthesis was studied by autoclaving these gum solutions (0.1–0.5%) containing 1 mM of silver nitrate for 30 min. Figure 2a shows the UV–Vis spectra of the produced silver nanoparticles with different concentrations of gum (0.1–0.5%) at 1 mM AgNO_3_ and 30 min of autoclaving. After autoclaving the silver nitrate containing gum solutions, the appearance of yellow color in the reaction mixtures was observed. This is a clear indication for the formation of silver nanoparticles by the gum. It reveals that the efficiency of nanoparticle synthesis increases with increasing concentration of gum. The synthesis was also evaluated by varying the reaction time (10–60 min) and reduction was studied with 0.5% gum at 1 mM AgNO_3_ (Figure 2b). It was noticed that the reduction capacity of the gum increased with reaction time. As the autoclaving time increases, possibly more and more of hydroxyl groups are being converted to carbonyl groups by air oxidation, which in turn reduce the silver ions. In the UV-Vis spectra a single strong peak with a maximum around 412 nm was observed, which corresponds to the typical surface plasmon resonance (SPR) of conducting electrons from the surface of silver nanoparticles. The SPR absorption of metal nanoparticles like gold and silver is very sensitive to the changes of the size and shape of the nanoparticles formed [29].

#### Transmission electron microscopy

**Figure 3:**
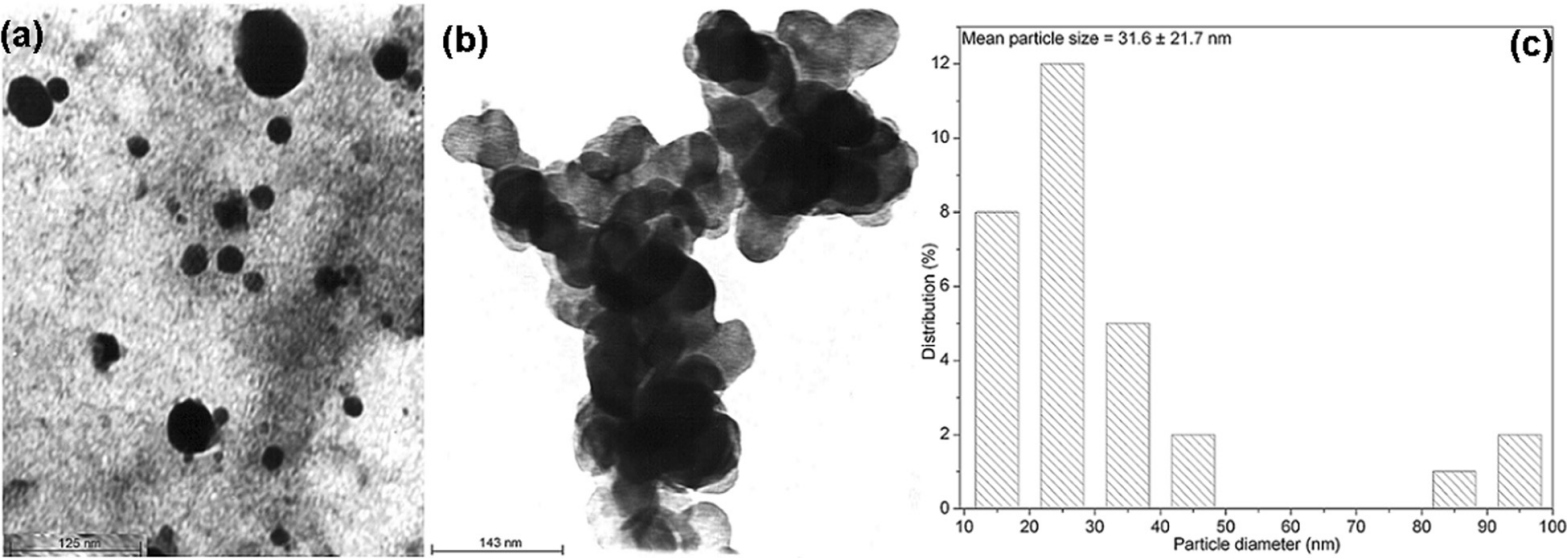
**TEM images of silver nanoparticles synthesized with 0.5% (w/v) gum ghatti and 1 mM AgNO**_**3**_**, autoclaved for 30 min, at (a) 125 nm, (b) 143 nm scale, and (c) histogram showing the particle size distribution.**

**Figure 4:**
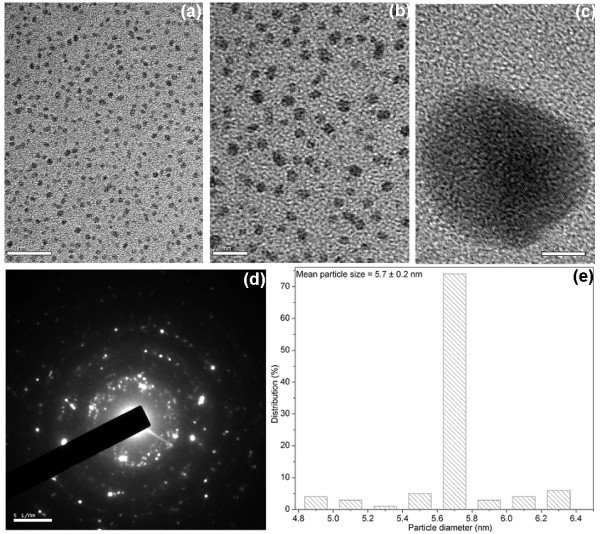
**TEM images of silver nanoparticles synthesized with 0.1% (w/v) gum ghatti and 1 mM AgNO**_**3**_**, autoclaved for 30 min, at (a) 50 nm, (b) 20 nm, and (c) 5 nm scale. (d) Corresponding SAED pattern and (e) histogram showing the particle size distribution.**

**Figure 5:**
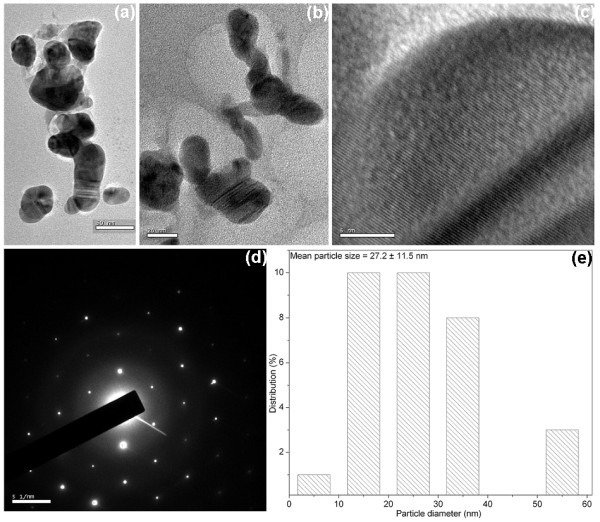
**TEM images of silver nanoparticles synthesized with 0.1% (w/v) gum ghatti and 1 mM AgNO**_**3**_**, autoclaved for 60 min, at (a) 50 nm, (b) 20 nm, and (c) 5 nm scale. (d) Corresponding SAED pattern and (e) histogram showing the particle size distribution.**

Figure 3 shows the TEM images of the silver nanoparticles synthesized with 0.5% gum and 1 mM AgNO_3_ autoclaved for 30 min. These nanoparticles are spherical, polydisperse, aggregated, and the average particle size obtained from these micrographs was about 31.6 ± 21.7 nm (Figure 3c). The influence of gum concentration on the morphology of the nanoparticles was investigated with 0.1% gum and 1 mM AgNO_3_, autoclaved for 30 min (Figure 4). These nanoparticles were spherical in shape and nearly isotropic in nature. The average particle size obtained from the corresponding diameter distribution was about 5.7 ± 0.2 nm (Figure 4e). The effect of autoclaving time on the shape and size of the nanoparticles was confirmed with 0.1% gum solution, autoclaved for 60 min at 1 mM AgNO_3_ (Figure 5). The TEM observations of this sample indicate the shape anisotropy and the nanoparticles display a rich variety of shapes in varying sizes. In addition to nanospheres, hexagonal, and polygonal nanoprisms, ellipsoidal and uneven shaped nanoparticles were observed. These nanoparticles are polydisperse, aggregated, and the average particle size obtained from these micrographs was about 27.2 ± 11.5 nm, for 60 min of reaction time (Figure 5e). The selected-area electron diffraction (SAED) patterns depicted in Figures 4d and 5d exhibit concentric rings with intermittent bright dots, indicating that these nanoparticles are highly crystalline in nature. These rings can be attributed to the diffraction from the (111), (200), (220), and (311) planes of face-centered cubic (fcc) silver. The crystallinity of the synthesized nanoparticles was also supported from the observed clear lattice fringes in high-resolution images (Figures 4c and 5c). Interestingly at 0.1% gum and 1 mM of AgNO_3_ concentration with 30 min of autoclaving, nearly 70% of the nanoparticles formed were in the size of 5.7 nm (Figure 4e). When the concentration of gum was decreased from 0.5 to 0.1%, the average particle size of the silver nanoparticles formed decreased. This was also confirmed in a previous study on size controllable synthesis of silver nanoparticles with tannic acid, in which the concentration of the polyphenol decreased from 23.5 to 1.8 μM [3]. The decrease in polydispersity with decrease in the concentration of gum was also evident from the TEM images (Figures 3 and 4). It is worth noting that the shape of the particles changed from spheres to anisotropic nanostructures, when the reaction time was increased to 60 min at 0.1% of gum concentration (Figures 4 and 5). This is most likely due to the continual growth of nanoparticles during longer period of autoclaving. This study indicates that the particle size of the silver nanoparticles can be controlled by varying the concentration of gum and reaction time. As a result, nanoparticles with near monodispersity were obtained with 0.1% gum and 30 min of reaction time at 1 mM of silver nitrate concentration.

#### X-ray diffraction

**Figure 6:**
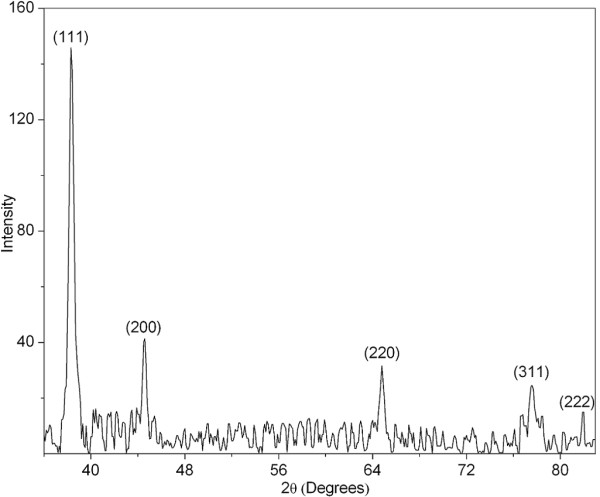
XRD pattern of the silver nanoparticles, indicating fcc crystal structure.

The XRD technique was used to determine and confirm the crystal structure of silver nanoparticles. The XRD pattern of the silver nanoparticles is shown in Figure 6. There were five well-defined characteristic diffraction peaks at 38.3°, 44.6°, 64.8°, 77.6°, and 81.9°, respectively, corresponding to (111), (200), (220), (311), and (222) planes of fcc crystal structure of metallic silver. The interplanar spacing (*d*_*hkl*_) values (2.348, 2.030, 1.437, 1.229, and 1.175 Å) calculated from the XRD spectrum of silver nanoparticles was in agreement with the standard silver values. Thus, the XRD pattern further corroborates the highly crystalline nature of nanoparticles observed from SAED patterns and high-resolution TEM images (Figures 4 and 5). The lattice constant calculated from this pattern was 4.061 Å, a value which is in agreement with the value reported in literature for silver (JCPDS PDF card 04–0783). Also, the broadening of the diffraction peaks was observed owing to the effect of nano-sized particles. As the nanoparticles are capped by the moieties of gum, the background observed was high.

#### Fourier transform infrared spectroscopy (FTIR)

**Figure 7:**
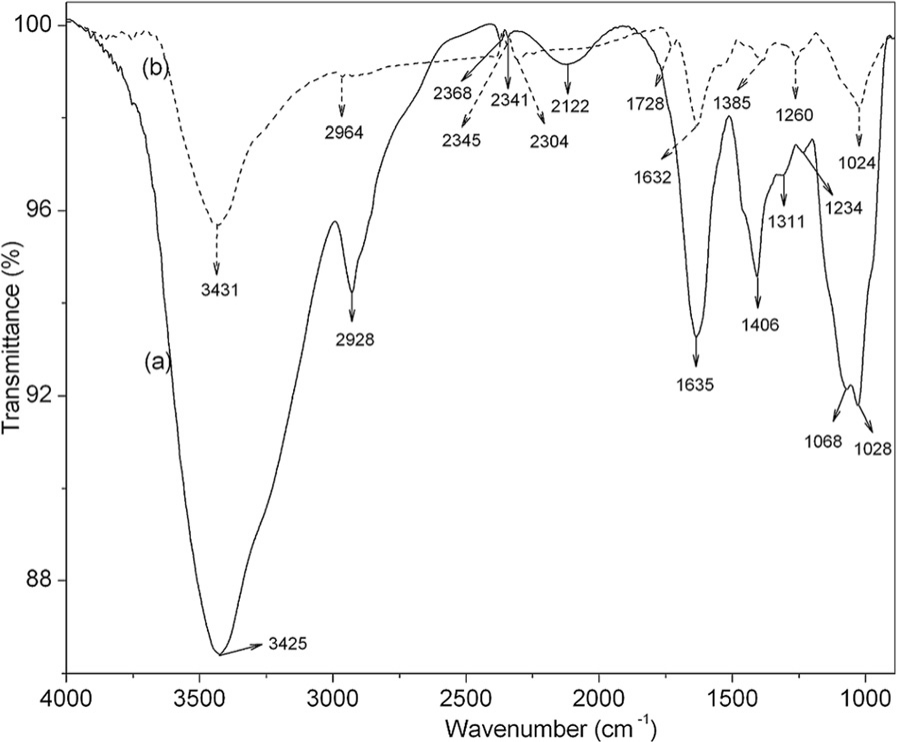
FTIR spectra of freeze dried (a) gum ghatti and (b) silver nanoparticles.

The FTIR spectra of the gum and nanoparticles were recorded in order to identify the functional groups of gum involved in the reduction and capping/stabilization of the synthesized nanoparticles. Figure 7 shows the FTIR spectra of the lyophilized gum and silver nanoparticles. The major absorbance bands present in the spectrum of gum ghatti were at 3425, 2928, 2368, 2341, 2122, 1635, 1406, 1311, 1234, 1068, and 1028 cm^−1^. The broad band observed at 3425 cm^−1^ could be assigned to stretching vibrations of O–H groups in gum ghatti. The bands at 2928, 1406, and 1234 cm^−1^ correspond to asymmetric stretching, scissoring; and twisting and rocking vibrations of methylene groups, respectively. The broad band at 2122 cm^−1^ only appeared in the spectrum of gum could be assigned to various carbonyl species. The stronger band found at 1635 cm^−1^ could be assigned to characteristic asymmetrical stretch of carboxylate group. The symmetrical stretch of carboxylate group can be attributed to the band present at 1311 cm^−1^. The peaks at 1068 and 1028 cm^−1^ were due to the C–O stretching vibration of ether and alcoholic groups, respectively [28]. While, the spectrum of lyophilized nanoparticles showed characteristic absorbance bands at 3431, 2964, 2345, 2304, 1728, 1632, 1385, 1260, and 1024 cm^−1^. In the IR spectrum of nanoparticles, a shift in the absorbance peaks was observed from 3425 to 3431 cm^−1^ and 1635 to 1632 cm^−1^, and 1311 to 1385 cm^−1^, suggesting the binding of silver ions with hydroxyl and carboxylate groups, respectively. It is pertinent to note that nanoparticles shows a new band at 1728 cm^−1^ corresponding to carbonyl stretching vibrations in aldehydes, ketones, and carboxylic acids [2]. Further, the occurrence of the peak at 1728 cm^−1^ and disappearance of the peak at 2122 cm^−1^ confirm that the reduction of the silver ions is coupled to the oxidation of the hydroxyl and carbonyl groups, indicative of more extensively oxidized nature of the gum. Based on the band shift in the hydroxyl and carbonyl groups and the loss of existing carbonyls and appearance of a new carbonyl peak, it can be inferred that both hydroxyl and carbonyl groups of gum are involved in the synthesis of silver nanoparticles. The variations in the shape and peak position of the hydroxyl and carboxylate groups have been reported, where silver nanoparticles were synthesized using another polysaccharide, gum *Acacia*[4].

#### Raman spectroscopy

**Figure 8:**
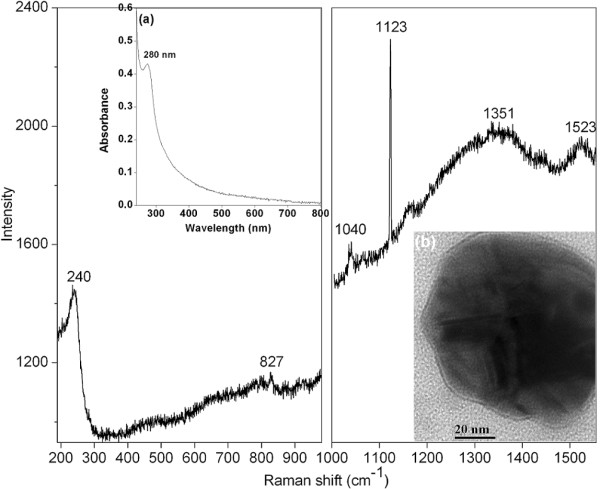
Raman spectrum of aqueous silver nanoparticle solution. (a) UV–Vis absorption spectrum of the 0.5% (w/v) gum solution against water blank, autoclaved for 30 min and (b) TEM image of a single nanoparticle, surrounded by a layer of organic matrix.

In order to find out the possible functional groups of capping agents associated in the stabilization of silver nanoparticles, Raman spectrum of the nanoparticles was recorded. Figure 8 gives the selective enhancement of Raman bands of the organic capping agents bound to the nanoparticles. The spectrum shows a strong and sharp band at 240 cm^−1^, which can be attributed to the stretching vibrations of Ag–N [30,31] and Ag–O bonds [32]. This peak indicates the formation of a chemical bond between silver and amino nitrogen [31]; and silver and carboxylate groups [32] of gum molecules. It confirms that the gum is bound to the silver nanoparticle surface either through amino or carboxylate group or both. It is known to have close frequencies for the Ag–N and Ag–O stretching vibrations and the involvement of both N and O atoms in binding result in surface-enhanced Raman scattering (SERS) band broadening [30]. The broad ones at 1351 and 1523 cm^–1^ correspond to symmetric and asymmetric C = O stretching vibrations of carboxylate group, respectively [31]. The enhancement in the intensity of the CO_2_ stretching vibration suggests the direct binding of the COO^−^ group with the silver surface [32]. The broad band at 1040 and a sharp peak at 1123 cm^–1^; the one at 827 cm^–1^ comes from the C–H in plane bending and out of plane wag, respectively [30], from the saccharide structure of gum. Thus, from the preferential enhancement of these bands; it can be concluded that both amino and carboxylate groups of the gum are involved in the capping of the silver nanoparticles. These results are in concurrence with earlier biosynthesis of silver nanoparticles carried out with non-pathogenic fungus *Trichoderma asperellum*[31]. It was reported earlier that the carboxylate groups of glycoprotein of gum *Acacia* were involved in binding of silver nanoparticles [4]. It is known that proteins can bind to nanoparticles either through free amino groups or by electrostatic interaction of negatively charged carboxylate groups [33]. The gum ghatti is known to contain protein and the protein content was reported to be in the range of 2.8–3.7% [13-17]. This observation is further substantiated by the measured protein concentration of 2.7% for the gum and the UV–Vis absorption spectrum of the 0.5% gum solution against water blank, autoclaved for 30 min, given in Figure 8a. An absorption peak at 280 nm is clearly visible and is attributed to electronic excitations in tryptophan and tyrosine residues in the proteins [1,33], which are present in the gum. The stabilization of nanoparticles by capping agents is also validated from the TEM image showing a single nanoparticle that is surrounded by a layer of organic matrix (Figure 8b). Thus, one can conclude that once the silver ions are reduced to silver nanoparticles by the polyhydroxylated gum, proteins present in the gum subsequently encapsulate and stabilize these particles along with saccharide molecules. Based on these observations, these silver nanoparticles can be used as a possible substrate for SERS. As observed in IR spectra (Figure 7), gum ghatti is rich in various functional groups; and their capping on silver nanoparticles provides surface reactivity. It is reported that the functional unit used as a capping agent plays an important role and determines the tissue distribution profile of gold nanoparticles [34]. Thus, these functionalized nanoparticles are useful for various applications such as drug delivery [6], targeted biological interactions [34], and biological labels [35].

#### Antibacterial assay

For checking the antibacterial activity, silver nanoparticles with an average size of 5.7 ± 0.2 nm were used. These nanoparticles were prepared with 0.1% gum solution containing 1 mM AgNO_3_, autoclaved for 30 min. After 24 h of incubation at 37°C, growth suppression was observed in plates loaded with 5 μg of silver nanoparticles. Whereas, the negative control plates loaded with autoclaved gum did not produce any ZOI. Gum–silver nanoparticles showed growth inhibition around the wells against the tested bacteria. ZOI of around 12.25 mm diameter was observed for the Gram-positive bacterial strain *S. aureus* ATCC 25923. In the case of Gram-negative bacterial strains *E. coli* ATCC 25922, *E. coli* ATCC 35218, and *P. aeruginosa* ATCC 27853, the detected ZOI were 9.0, 8.0, and 11.0 mm, respectively. As expected, the positive control plates loaded with silver nitrate exhibited inhibition zones (Table 1). The ZOI values noted for different bacterial strains with silver nanoparticles are comparable with the positive controls. Based on these results, it can be concluded that the synthesized silver nanoparticles had significant antibacterial action on both the Gram classes of bacteria.

**Table 1:** Inhibition zones (mm) observed with different bacterial culture plates loaded with silver nanoparticles and silver nitrate at a silver concentration of 5 μg

**Test compound**	***S. aureus*25923**	***E. coli*25922**	***E. coli*35218**	***P. aeruginosa*27853**
Silver nanoparticles	12.25	9.0	8.0	11.0
Silver nitrate	13.5	11.0	7.6	12.0

## Experimental

### Synthesis of silver nanoparticles

Silver nitrate (AgNO_3_) (E. Merck, Mumbai, India) of analytical reagent grade was used for the synthesis. “Gum ghatti” grade-1 was purchased from Girijan Co-operative Corporation Ltd., Hyderabad, India. All the solutions were prepared in ultra pure water. Gum ghatti was powdered in a Prestige high-speed mechanical blender (Bengaluru, India) and sieved to obtain a mean particle size of 38 μm. Then, 0.5% (w/v) of homogenous gum stock solution was prepared by adding this powder to reagent bottle containing ultra pure water and stirring overnight at room temperature. Then this solution was centrifuged to remove the insoluble materials and the supernatant was used for all the experiments. The protein concentration in the gum solution was quantified by Lowry’s method using a Bangalore Genei^TM^ protein estimation kit, Cat No 105560 (Bengaluru, India). The silver nanoparticles were synthesized by autoclaving the silver nitrate solutions containing various concentrations of gum ghatti at 121°C and 103 kPa of pressure for different durations of time, under dark conditions. The effect of concentration of gum and reaction time on nanoparticle synthesis was studied.

### Antibacterial assay

The well-diffusion method was used to study the antibacterial activity of the synthesized silver nanoparticles. All the glassware, media, and reagents used were sterilized in an autoclave at 121°C, 103 kPa of pressure for 20 min. *Staphylococcus aureus* (ATCC 25923); and *Escherichia coli* (ATCC 25922), *E. coli* (ATCC 35218), and *Pseudomonas aeruginosa* (ATCC 27853) were used as model test strains for Gram-positive and Gram-negative bacteria, respectively. Bacterial suspension was prepared by growing a single colony overnight in nutrient broth and by adjusting the turbidity to 0.5 McFarland standard [36]. Mueller Hinton agar plates were inoculated with this bacterial suspension and 5 μg of silver nanoparticles was added to the center well with a diameter of 6 mm. The nanoparticles used were prepared with 0.1% gum solution containing 1 mM AgNO_3_, autoclaved for 30 min. Negative control plates were maintained with autoclaved gum-loaded wells. The culture plates loaded with silver nitrate at a silver concentration of 5 μg were included as positive controls. These plates were incubated at 37°C for 24 h in a bacteriological incubator and the zone of inhibition (ZOI) was measured by subtracting the well diameter from the total inhibition zone diameter. Three independent experiments were carried out with each bacterial strain.

## Conclusions

This study reports the facile synthesis of silver nanoparticles from silver nitrate using gum ghatti. The adopted method is compatible with green chemistry principles as the gum serves as a dual functional reductant and stabilizer for the synthesis of nanoparticles. At a given gum concentration, the efficiency of nanoparticle synthesis increases with reaction time, a property attributable to the large reduction capacity of the gum. As the particle size of the nanoparticles can be controlled, this method can be implemented for the large-scale production of monodispersed and spherical nanoparticles of around 5.7 nm due to the availability of low-cost plant-derived biopolymer**.** The hydroxyl and carboxylate groups of the gum facilitate the complexation of silver ions during autoclaving. Subsequently, these silver ions are reduced to elemental silver possibly by *in situ* oxidation of hydroxyl groups; and by the intrinsic carbonyl groups in addition to those produced by the air oxidation. This proposed mechanism is also substantiated by the FTIR data. Further, the formed silver nanoparticles had significant antibacterial action on both the Gram classes of bacteria. The surface reactivity provided by capping enables these functionalized nanoparticles as promising candidates for various pharmaceutical, biomedical, and environmental applications. Notably, the selective enhancement of Raman bands of the organic capping agents bound to the silver colloids allows these nanoparticles as suitable substrates for SERS. In view of this, further studies are envisaged to explore the other potential applications of this gum-based nanoparticles.

## Competing interests

The authors declare that they have no competing interests.

## References

[B1] Vigneshwaran N Nachane RP Balasubramanya RH Varadarajan PV A novel one-pot ‘green’ synthesis of stable silver nanoparticles using soluble starch Carbohydr Res 2006 341 2012 2018 10.1016/j.carres.2006.04.042 16716274

[B2] Wei D Qian W Facile synthesis of Ag and Au nanoparticles utilizing chitosan as a mediator agent Colloids Surf B 2008 62 136 142 10.1016/j.colsurfb.2007.09.030 17983734

[B3] Dadosh T Synthesis of uniform silver nanoparticles with a controllable size Mater Lett 2009 63 2236 2238 10.1016/j.matlet.2009.07.042

[B4] Mohan YM Raju KM Sambasivudu K Singh S Sreedhar B Preparation of acacia-stabilized silver nanoparticles: a green approach J Appl Polym Sci 2007 106 3375 3381 10.1002/app.26979

[B5] Kora AJ Sashidhar RB Arunachalam J Gum kondagogu (Cochlospermum gossypium): a template for the green synthesis and stabilization of silver nanoparticles with antibacterial application Carbohydr Polym 2010 82 670 679 10.1016/j.carbpol.2010.05.034

[B6] Dhar S Reddy EM Shiras A Pokharkar V Prasad BLV Natural gum reduced/stabilized gold nanoparticles for drug delivery formulations Chem Eur J 2008 14 10244 10250 10.1002/chem.200801093 18850613

[B7] Panda H Gum ghatti The complete technology book on natural products (Forest based) 2003 National Institute of Industrial Research, Delhi 1 9

[B8] Nussinovitch A Miscellaneous uses of plant exudates Plant gum exudates of the world: sources, distribution, properties and applications 2010 CRC Press, Taylor and Francis, Boca Raton, USA 347 368

[B9] Srivastava VK Rai RS Physico-chemical studies on gum Dhawa (Anogeissus latifolia wall.) Colloid Polym Sci 1963 190 140 143 10.1007/BF01513532

[B10] Aspinall GO Bhavanadan VP Christensen TB Gum ghatti (Indian gum). Part V. Degradation of the periodate-oxidised gum J Chem Soc 1965 2677 2684

[B11] Jefferies M Pass G Phillips GO Viscosity of aqueous solutions of gum ghatti J Sci Food Agric 1977 28 173 179 10.1002/jsfa.2740280211

[B12] Tischer CA Iacomini M Wagner R Gorin PAJ New structural features of the polysaccharide from gum ghatti (Anogeissus latifola) Carbohydr Res 2002 337 2205 2210 10.1016/S0008-6215(02)00296-3 12433484

[B13] Amar V Al-Assaf S Phillips GO An introduction to gum ghatti: another proteinaceous gum Foods Food Ingredients J Jpn 2006 211 275 280

[B14] Katayama T Ido T Sasaki Y Ogasawara T Al-Assaf S Phillips GO Characteristics of the adsorbed component of gum ghatti responsible for its oil–water interface advantages Foods Food Ingredients J Jpn 2008 213 372 376

[B15] Ido T Ogasawara T Katayama T Sasaki Y Al-Assaf S Phillips GO Emulsification properties of GATIFOLIA (Gum ghatti) used for emulsions in food products Foods Food Ingredients J Jpn 2008 213 365 371

[B16] Kaur L Singh J Singh H Characterization of gum ghatti (Anogeissus latifolia): a structural and rheological approach J Food Sci 2009 74 E328 E332 10.1111/j.1750-3841.2009.01244.x 19723196

[B17] Castellani O Gaillard C Vié V Al-Assaf S Axelos M Phillips GO Anton M Hydrocolloids with emulsifying capacity. Part 3: adsorption and structural properties at the air–water surface Food Hydrocolloids 2010 24 131 141 10.1016/j.foodhyd.2009.07.009

[B18] Shahane J Dinkache ladu-remembrance of things past http://thecookscottage. typepad.com/curry/2006/03/dinkache_ladure.html Accessed 17 March 2010

[B19] Meena KL Yadav BL Some ethnomedicinal plants of southern Rajasthan Indian J Tradit Knowl 2010 9 169 172

[B20] Crociani F Alessandrini A Mucci MM Biavati B Degradation of complex carbohydrates by Bifidobacterium spp Int J Food Microbiol 1994 24 199 210 10.1016/0168-1605(94)90119-8 7703014

[B21] Hill MJ Bacterial fermentation of complex carbohydrate in the human colon Eur J Cancer Prevent 1995 4 353 358 10.1097/00008469-199510000-00004 7496323

[B22] Ramberg J Gardiner T Ghatti gum—Anogeissus latifolis stem gum (ghatti gum). GlycoEssential 7 Ingredients http://www.glyconutrients-center.org/ghatti-gum.php. Accessed 17 March 2010

[B23] Mishra A Raikwar A Anogeissus latifolia (ghatti gum): the edible gum Vaniki Sandesh 2005 29 27 28

[B24] Fahrenbach MJ Riccardi BA Grant WC Hypocholesterolemic activity of mucilaginous polysaccharides in white leghorn cockerels Proc Soc Exp Biol Med 1966 123 321 326 5924467 10.3181/00379727-123-31478

[B25] Parvathi KMM Ramesh CK Krishna V Paramesha M Kuppast IJ Hypolipidemic activity of gum ghatti of Anogeissus latifolia Phcog Mag 2009 5 11 14

[B26] Joshi MG Setty CM Deshmukh AS Bhatt YA Gum ghatti: a new release modifier for zero-order release in 3-layered tablets of diltiazem hydrochloride Indian J Pharm Educ Res 2010 44 78 85

[B27] Raveendran P Fu J Wallen SL Completely “green” synthesis and stabilization of metal nanoparticles J Am Chem Soc 2003 125 13940 13941 10.1021/ja029267j 14611213

[B28] Edwards HGM Falk MJ Sibley MG Alvarez-Benedi J Rull F FT-Raman spectroscopy of gums of technological significance Spectrochim Acta A 1998 54 903 920 10.1016/S1386-1425(98)00018-3

[B29] Huanga NM Lim HN Radiman S Khiew PS Chiu WS Hashim R Chia CH Sucrose ester micellar-mediated synthesis of Ag nanoparticles and the antibacterial properties Colloids Surf A 2010 353 69 76 10.1016/j.colsurfa.2009.10.023

[B30] Chowdhury J Ghosh M Concentration-dependent surface-enhanced Raman scattering of 2-benzoylpyridine adsorbed on colloidal silver particles J Colloid Interface Sci 2004 277 121 127 10.1016/j.jcis.2004.04.030 15276048

[B31] Mukherjee P Roy M Mandal BP Dey GK Mukherjee PK Ghatak J Tyagi AK Kale SP Green synthesis of highly stabilized nanocrystalline silver particles by a non-pathogenic and agriculturally important fungus T. asperellum Nanotechnology 2008 19 075103 075109 10.1088/0957-4484/19/7/075103 21817628

[B32] Biswas N Kapoor S Mahal HS Mukherjee T Adsorption of CGA on colloidal silver particles: DFT and SERS study Chem Phys Lett 2007 444 338 345 10.1016/j.cplett.2007.07.049

[B33] Vigneshwaran N Ashtaputre NM Varadarajan PV Nachane RP Paralikar KM Balasubramanya RH Biological synthesis of silver nanoparticles using the fungus Aspergillus flavus Mater Lett 2007 61 1413 1418 10.1016/j.matlet.2006.07.042

[B34] Genevieve MF Stan WC Dae Young K Raghuraman K Kavita K Nripen C Kattesh K Biodistribution of maltose and gum arabic hybrid gold nanoparticles after intravenous injection in juvenile swine Nanomed: Nanotech Biol Med 2009 5 128 135 10.1016/j.nano.2009.01.007 19480048

[B35] Schrand AM Braydich-Stolle LK Schlager JJ Dai L Hussain SM Can silver nanoparticles be useful as potential biological labels? Nanotechnology 2008 19 235104 235116 10.1088/0957-4484/19/23/235104 21825779

[B36] Kora AJ Manjusha R Arunachalam J Superior bactericidal activity of SDS capped silver nanoparticles: Synthesis and characterization Mater Sci Eng C 2009 29 2104 2109 10.1016/j.msec.2009.04.010

